# An Automated High-Throughput Screening (HTS) Spotter for 3D Tumor Spheroid Formation

**DOI:** 10.3390/ijms24021006

**Published:** 2023-01-05

**Authors:** Mi-Hyeon Jeong, Inhee Kim, Kyunghyun Park, Bosung Ku, Dong Woo Lee, Kyoung Ryeol Park, Sang Youl Jeon, Jung Eun Kim

**Affiliations:** 1 Central R & D Center, Medical & Bio Decision (MBD) Co., Ltd., Suwon-si 16229, Gyeonggi-do, Republic of Korea; 2 Department of Biomedical Engineering, Gachon University, Seongnam-si 13120, Gyeonggi-do, Republic of Korea

**Keywords:** bioprinting-based dispenser, 3D spheroids/organoids, miniaturization, high-throughput screening (HTS)

## Abstract

Three-dimensional (3D) culture platforms have been adopted in a high-throughput screening (HTS) system to mimic in vivo physiological microenvironments. The automated dispenser has been established commercially to enable spotting or distributing non-viscous or viscous biomaterials onto microplates. However, there are still challenges to the precise and accurate dispensation of cells embedded in hydrogels such as Alginate- and Matrigel-extracellular matrices. We developed and improved an automated contact-free dispensing machine, the ASFA SPOTTER (V5 and V6), which is compatible with 96- and 384-pillar/well plates and 330- and 532-micropillar/well chips for the support of 3D spheroid/organoid models using bioprinting techniques. This enables the distribution of non-viscous and viscous biosamples, including chemical drugs and cancer cells, for large-scale drug screening at high speed and small volumes (20 to 4000 nanoliters) with no damage to cells. The ASFA SPOTTER (V5 and V6) utilizes a contact-free method that minimizes cross-contamination for the dispensation of encapsulated tissue cells with highly viscous scaffolds (over 70%). In particular, the SPOTTER V6 does not require a washing process and offers the advantage of almost no dead volume (defined as additional required sample volume, including a pre-shot and flushing shot for dispensing). It can be successfully applied for the achievement of an organoid culture in automation, with rapid and easy operation, as well as miniaturization for high-throughput screening. In this study, we report the advantages of the ASFA SPOTTER, which distributes standard-sized cell spots with hydrogels onto a 384-pillar/well plate with a fast dispensing speed, small-scale volume, accuracy, and precision.

## 1. Introduction

The additive manufacturing of biofabrication has emerged as a high-throughput bioprinting technique for spatial patterning on surfaces, using bio-inks consisting of living cells and non-living materials (scaffold-based or scaffold-free) [1,2,3,4]. Bioprinting technology includes inkjet-, acoustic-, and micro-solenoid valve-based methods [3,4,5]. Micro-solenoid valve-based bioprinting is preferable to other methods for the ejection of biosamples containing highly viscous materials, such as Alginate, Matrigel, and basement membrane extract (BME), and it enables the growth of cells in three dimensions (3D) with no damage. Cell death caused by heat can occur as a result of inkjet bioprinting, and acoustic bioprinting is not applicable for the ejection of viscous samples with high-density cells [3,4,6]. A micro-solenoid valve, comprising of a plunger, solenoid coil, core spring, and orifice, controls the time that the nozzle orifice is open with an applied voltage pulse. It enables the printing of viscous biosamples, including cells within hydrogels, onto a conventional microwell plate and micropillar chip [7,8].

Automated miniaturization techniques for 3D spheroid/organoid models are indispensable workstations for the manipulation of a small volume of biomaterials (living cells in hydrogels) with uniform size for architecture mimicking physical microenvironments and for the identification of new drug candidates [9,10,11,12]. Many pharmaceutical and bio companies have made efforts to develop robotic liquid dispensers using bioprinter technologies for the precise and accurate distribution of cells. However, there are some limitations in dispensing cells encapsulated in high-viscosity hydrogels without damage. In addition, they have difficulties in handling small scales and guaranteeing accurate cellular microenvironments [7,12,13,14]. In an effort to overcome these challenges and to obtain high-resolution results, we developed a micro-solenoid bioprinting-based dispensing machine, the ASFA SPOTTER (V5 and V6), which has the capacity to perform the automated printing of living cells in hydrogels onto the surface of 96- and 384-pillar/well plates and 330- and 532-micropillar/well chips. We confirmed that cells embedded in Alginate, Matrigel, or BME-based extracellular matrices were distributed at a standard size or volume (small scale; 20 to 4000 nanoliters) and were able to self-assemble and proliferate in 3D via ASFA SPOTTER. It is the most suitable applicator for spotting droplets of cancer cells with rapid speed, miniaturization, precision, and accuracy for the high-throughput screening of drug candidates.

## 2. Materials and Methods

### 2.1. The Automated Cell Dispenser, ASFA SPOTTER, for HTS

Bioprinting applicators are utilized for printing biomaterials on multiple-well plates, and several bio companies have developed automated dispensers to provide better 3D printing techniques [15]. The conventional automatic pipette [16] and spotter [17] are suitable to dispense biosamples at easy-to-operate and high speeds with microliter volumes; however, there may be some limitations in the performance of large-volume dispensing and sample loss from the washing step (Table 1). The advanced use of 3D spheroid/organoid models is required for the screening and development of drug candidates in preclinical and clinical fields. Accordingly, a dispensing machine is required for the accurate and precise development of supporting miniaturized organoid platforms.

**Figure 1:**
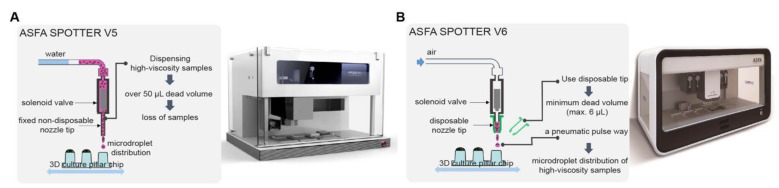
The scheme of ASFA Spotters. (**A**) ASFA Spotter V5. (**B**) ASFA Spotter V6.

The ASFA SPOTTERs (V5 and V6) were programmed for dispensing 20 to 4000 nanoliter volumes of non-viscous and viscous biosamples on 96- and 384-pillar/well plates and 330- and 532-micropillar/well chips. They are fundamentally composed of a micro-solenoid valve for determining the dispensing volume according to the valve open time, a nozzle acting as a gate for cells, a source plate, and a target plate, and they are capable of spotting cell droplets within 60 s per 384-pillar plate. To support growth in 3D, these applicators print cancer cells and growth medium with various scaffolds, such as hydrogels, on a micropillar/well chip; micropillar/well chips are sandwiched in microwell plates containing valid growth medium, which then allows cancer cells to aggregate by gravity forming cancer spheroids/organoids [18,19]. The ASFA SPOTTER V5 [20] (Figure 1A and Video S1) utilizes water pressure to manipulate the nozzle orifice and a non-disposable stainless nozzle for dispensing biosamples. To minimize clogging issues by cells or high-viscosity materials, biosamples should be well-suspended by pipetting, and the micro-solenoid valve and aspiration tubing should be pre-chilled with cold water. It can distribute 20 to 4000 nanoliter scales per spot of encapsulated cells with extracellular matrices (Alginate, BME, and Matrigel) within a second by free contact, and is configured with one source plate and two target plates for automated application (Table 1). However, there are drawbacks in the process of dispensing cells; the samples are mixed with water after aspiration into the tubing and are lost. Therefore, it requires over 50 microliters more than the actual volume to dispense samples onto the surface of pillar plates. A non-disposable stainless nozzle allows the dispensing of only one kind of sample at a time, and time is required for replacement with other samples due to washing (Table 1). In addition, contamination can occur during the sample removal (washing out) and aspiration into the tubing.

The ASFA SPOTTER V6 [21] (Figure 1B and Video S2) was developed to complement the shortcomings of V5. It is composed of a micro-solenoid valve, a syringe pump for the aspiration of biosamples from a source plate, an electro-pneumatic regulator, and a disposable nozzle tip. The V6 utilizes air instead of water as a pressure source for the manipulation of the dispensed sample volume using an electro-pneumatic regulator. Unlike V5, an advantage of application of a disposable plastic nozzle tip is that washing of tubing with water is not required and there is no cross-contamination between samples. Biosamples, including cancer cells, in highly viscous materials (hydrogels) are directly added to the disposable tip, and chilling is performed in a work manifold to avoid unwanted clogging, such as hydrogel gelation. Therefore, unnecessary sample loss is reduced by dispensing cells in hydrogels with the disposable tip onto the surface of a pillar plate or micropillar chip; only an additional 6 microliters per nozzle tip is required. Dispensing time (within a second per spot) is also reduced because no washing is required, and the SPOTTER V6 can be programmed for simultaneous dispensing using 4 different disposable nozzle tips and is configured with 1 source plate and 3 target plates (Table 1).

### 2.2. Dispensing Accuracy and Precision

Orange G dye (#O3567, Sigma, St. Louis, USA) was used for determining accuracy and precision after dispensing 0.5% Alginate (#W201502, Sigma, St. Louis, USA), 50% Matrigel (#354230, Corning, St. Louis, USA), and 70% BME (#3536-005-002, R&D systems, MN, USA) on a 384-well plate (#38384, SPL life science, Gyeonggi-do, Republic of Korea), with 20 to 4000 nanoliters per well. The volume of the distributed samples was confirmed with an absorbance of 450 nm after shaking at room temperature for 40 min.

A549 cells stably expressing green fluorescent protein (GFP) were used to verify accuracy and precision. An amount of 2.5 × 10^3^ cells per spot were embedded with 50% Matrigel and 70% BME, diluted with high glucose DMEM (#11995-065, Thermo Fisher Scientific, MA, USA) supplemented with 10% fetal bovine serum DMEM (#16000-044, Thermo Fisher Scientific, MA, USA) and 1% penicillin/streptomycin (#30-002-CI, Corning, NY, USA). The ASFA SPOTTERs V5 and V6 (MBD, Gyeonggi-do, Republic of Korea) were programmed for dispensing 20 to 4000 nanoliters per spot on the top of a 384-pillar plate (#MBD-PM384, MBD, Gyeonggi-do, Republic of Korea). The measurement of the green fluorescent signal was performed using an ASFA Scanner (MBD, Gyeonggi-do, Republic of Korea), and analysis was then performed using an ASFA Analyzer (MBD, Gyeonggi-do, Republic of Korea) for the calculation of the cell area. CellTiter-Glo (#9683, Promega, Madison, USA) was used for luminescent cell viability, following the manufacturer’s protocol.

## 3. Results

**Figure 2:**
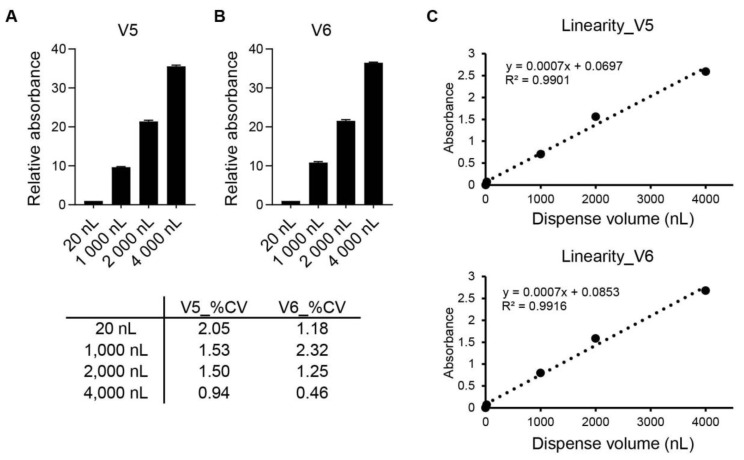
Dispense accuracy and precision of determined volumes. (**A**,**B**) 70% BME with Orange G dye was dispensed with indicated volume into 20 wells of 384-well plates using ASFA SPOTTERs V5 (**A**) and V6 (**B**). The absorbance was confirmed at 450 nm for dispensing accuracy and precision. (**C**) The absorbance mean values at each volume were plotted for linear regression.

The accuracy and precision of dispensing by ASFA SPOTTERs V5 and V6 were determined at volumes of 20, 1000, 2000, and 4000 nanoliters per well of 384-well plates using 70% BME. As shown in Figure 2A,B, the ASFA SPOTTERs V5 and V6 can accurately dispense the determined volumes of sample into the well plates as confirmed by absorbance; the precision of the coefficient of variance (%CV) was very good, with less than 3% at the determined volumes. In addition, the linearity of dispense was observed when the mean value of determined volumes was plotted. The linear regression coefficient (R^2^) was good, and it was high relative to the mean (R^2^ = 0.99) (Figure 2C).

**Figure 3:**
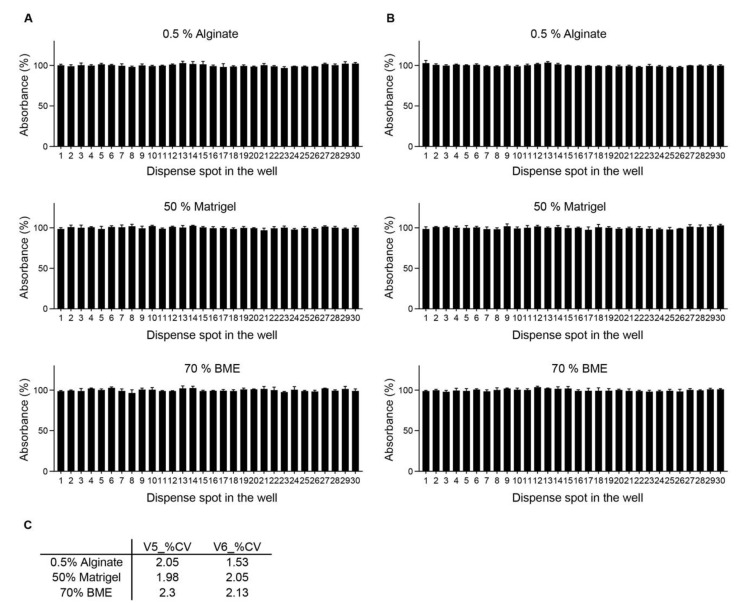
Dispensing accuracy and precision of ASFA SPOTTERs V5 and V6 when spotting various materials. (**A**,**B**) The high-viscosity biomaterials (0.5% Alginate, 50% Matrigel, and 70% BME) were dispensed into the 384-well plates with Orange G dye using ASFA SPOTTERs V5 (**A**) and V6 (**B**). The absorbance at 450 nm was measured for determination of the dispensing accuracy and precision. (**C**) The coefficient of variation (%CV) was calculated by dividing the mean absorbance by the standard deviation and then multiplying it by 100.

To determine the dead volume (additional required volume, including a pre-shot and flushing shot) in conditions with 0.5% Alginate, 50% Matrigel, and 70% BME, the 72 microliters of viscous solution were dispensed with 48 spots into the well plate (1500 nanoliters per spot) using ASFA SPOTTER V6. Consistent dispensing of biomaterials up to 44 spots for BME and Matrigel and 47 spots for Alginate was achieved, as demonstrated by absorbance. It demonstrates that 6 microliters for BME and Matrigel and 1.5 microliters for Alginate are additionally required for dispensing biosamples with a similar scale, with no need for the pre-shot and flushing shot (Figure S1A–C). When determining dead volume for ASFA SPOTTER V5, over 50 microliters is required for dispensing a viscous solution with a similar scale, including a pre-shot and flushing shot. Next, the dispense accuracy and precision were confirmed when spotting various materials using ASFA SPOTTERs V5 and V6 (Figure 3A,B). For these experiments, the biomaterials (0.5% Alginate, 50% Matrigel, and 70% BME) were dispensed in 30 wells, in three independent experiments. The dispensing accuracy was good using ASFA SPOTTERs V5 and V6, as confirmed by absorbance, demonstrating that the ASFA SPOTTER can distribute biosamples with a standard volume. In addition, the dispensing precision, as determined by the coefficient of variance (%CV), was very good, less than 3% (Figure 3C). These results present that the ASFA SPOTTER can be applied for dispensing high-viscosity biosamples with a consistent volume.

**Figure 4:**
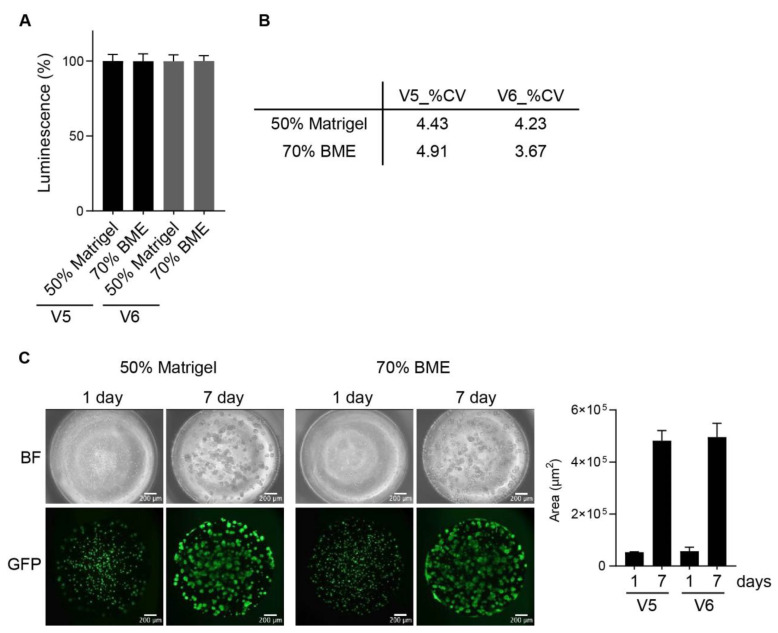
Applicable to growth in 3D. (**A**) A549 cells expressing GFP were dispensed with 50% Matrigel and 70% BME via ASFA SPOTTERs V5 and V6. Luminescence was then observed to confirm dispensing accuracy and precision. (**B**) The coefficient of variation (%CV) was calculated by dividing the mean value of luminescence by the standard deviation and then multiplying it by 100. (**C**) Cells were grown for a week after being dispensed onto the 384-pillar plates, and observation and analysis were then performed using an ASFA Analyzer.

A549 cells expressing GFP embedded in hydrogels (50% Matrigel and 70% BME) were dispensed onto the surface of a 384-pillar plate to verify the accuracy and precision of cell distribution with no clogging using ASFA SPOTTERs V5 and V6. The dispensed cells were analyzed by luminescence, cell volume was uniform, and the precision (%CV) was excellent, in that it composed less than 5% in all tested experiments (Figure 4A,B). Next, we confirmed cell growth distributed by the ASFA SPOTTER, and dispensed cells were self-assembled and proliferated to form 3D tumor spheroids (Figure 4C). This shows that the ASFA SPOTTER (V5 and V6) has advantages for dispensing cells with a high-viscosity solution and for the growth of cells in 3D with no damage.

## 4. Discussion

The bioprinting technique has emerged as a high-throughput screening to support 3D cultures for drug discovery and disease modeling in the academic and industrial fields [1,2,22]. The existing bioprinting platforms, including inkjet- and acoustic-based methods, have become widespread for 3D printing biomaterials with cells; however, there are some limitations, including low print efficiency, the inability to use high-viscosity biomaterials, and cell damage [15,22]. The use of micro-solenoid valve-based bioprinting, which can overcome the issue of cell viability and the availability of high-viscosity or high-density biomaterials, is a preferable approach to use of the inkjet- and acoustic-based methods; however, there are still challenges, including relatively slow speed, low resolution, and larger droplets [8,23].

The ASFA SPOTTERs (V5 and V6) are programmed for automated dispensation with a small-scale range of up to three target plates for supporting 3D spheroid/organoid models for the screening of large-scale drug candidates by using a micro-solenoid valve-based bioprinting strategy. In addition, they utilize a free-contact way for the prevention of cross-contamination and for dispensing biosamples with rapid speed to save valuable time. In particular, the ASFA SPOTTER V6 complements the drawbacks of V5 by using a disposable nozzle tip; it does not require a washing step and has the benefits of less sample loss and almost no dead volume. As shown in Figure 4, cancer cells were dispensed in uniform size with no damage, and it enables self-assembly and proliferation. The dispensing accuracy and precision of dispensing viscosity solution with or without cancer cells were high when estimated by absorbance, GFP signal, and luminescence. The ASFA SPOTTER can be extensively applied for the assessment of high-throughput screening with miniaturization, accuracy, and precision for biosample droplets.

The tumor spheroid/organoid model system has been adopted to assist in preclinical or clinical drug screening and decision making by clinicians with regard to treatment [24]. In accordance with this, the bioprinting application has been applied to provide more advanced and more rapid uniform formation of 3D spheroids/organoids and for the prediction of drug response and radiosensitivity for individual patients [15,25]. The ASFA SPOTTER supports a platform for successful spheroid/organoid formation by dispensing cancer cells with various scaffolds with high print efficacy. Printed cancer cells have the capacity for growth in 3D on micropillar/well chips and have been tested in several chemo drugs with various concentrations [21] and radiation responses [26]. This 3D bioprinting application provides accelerated drug screening, development, and precision medicine for effective therapeutic decision making in clinical practice.

## 5. Conclusions

The main points of the ASFA SPOTTER are as follows:

- It is composed of a micro-solenoid valve, syringe pump, electro-pneumatic regulator (for V6), nozzle tips, source plate, and target plate.
- It is programmed for dispensing biomaterials with various volumes at nL to μL, with high accuracy and precision.
- The SPOTTER V6 complements the weaknesses (large dead volume, sample dilution, cross-contamination, etc.) of V5 by the application of disposable nozzle tips.
- It can dispense high-viscosity biomaterials with high accuracy and precision.
- It enables self-assembly and the growth of cells in 3D on the micropillar/well chip.
- Good resolution results can be obtained by supporting miniaturized organoid platforms.
- It can support the optimization of drug screening and the selection of an effective treatment in order to improve therapeutic outcomes by the utilization of easy-to-use and rapid in vitro platforms for 3D cultures.

## Figures and Tables

**Table 1:** The performance comparison between conventional dispensers and ASFA SPOTTERs V5 and V6.

	Conventional Automatic Pipette (Biomek i5) 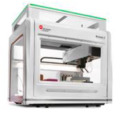	Conventional Spotter (BioDot Sphera^TM^ Platform) 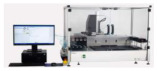	ASFA Spotter V5 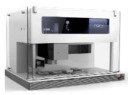	ASFA Spotter V6 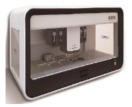
Pressure source	-	water	water	air
Target plate number	-	-	2	3
Nozzle tip type	disposable	fixed non-disposable	fixed non-disposable	disposable
Nozzle number			max. 6	max. 4
Dispensing volume	0.5–1000 μL	2–30 μL	20–4000 nL	20–4000 nL
Dead volume	-	-	over 50 μL	max. 6 μL
Washing step	not necessary	necessary	necessary	not necessary
Features	It can dispense large-volume biomaterials Easily automated system	High speed Non-contact dispensing	No clogging issues High cell viability Non-contact dispensing It can print high viscosity biomaterials	No clogging issues High cell viability It can print high viscosity biomaterials No sample dilution It prevents cross-contamination between samples Save valuable time to dispense biomaterials Fast and easy to operate High printing efficiency
limitations	It may not be applicapable to dispense with low-scale volume	It may be possble to sample loss by washing step	Sample loss by washing step It is possible to cross-contamination	-

## Data Availability

Not applicable.

## Supplementary Materials

The following supporting information can be downloaded at: https://www.mdpi.com/article/10.3390/ijms24021006/s1.
