# Supplementary figures and images for: An Automated High-Throughput Screening (HTS) Spotter for 3D Tumor Spheroid Formation
Source: Int J Mol Sci. 2023 Jan 5;24(2):1006. doi: 10.3390/ijms24021006 (PMC9867480; doi:10.3390/ijms24021006)

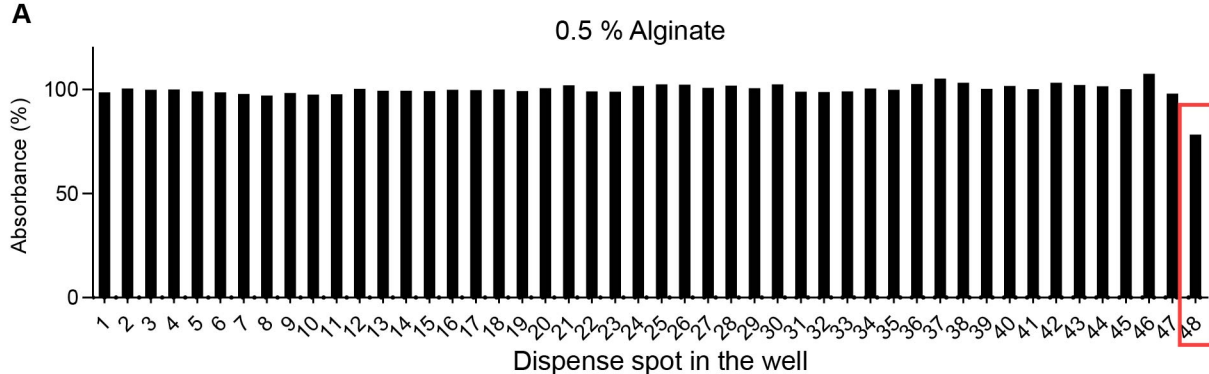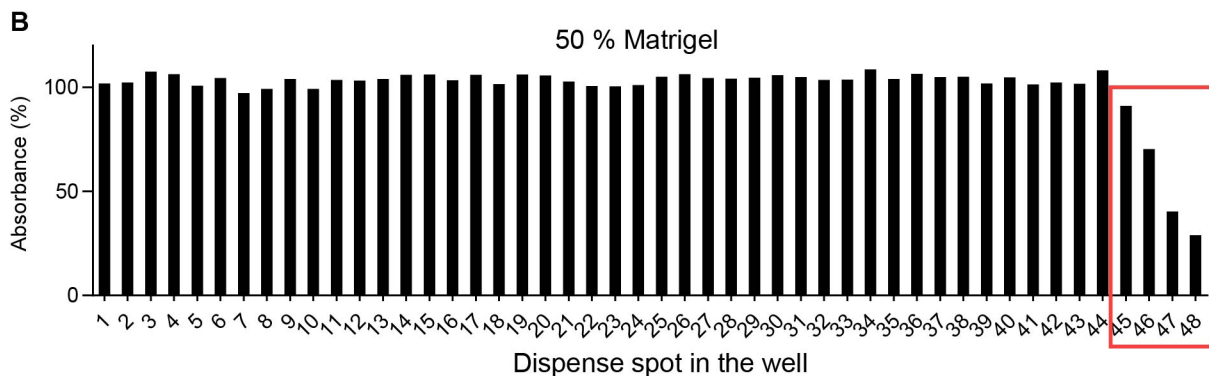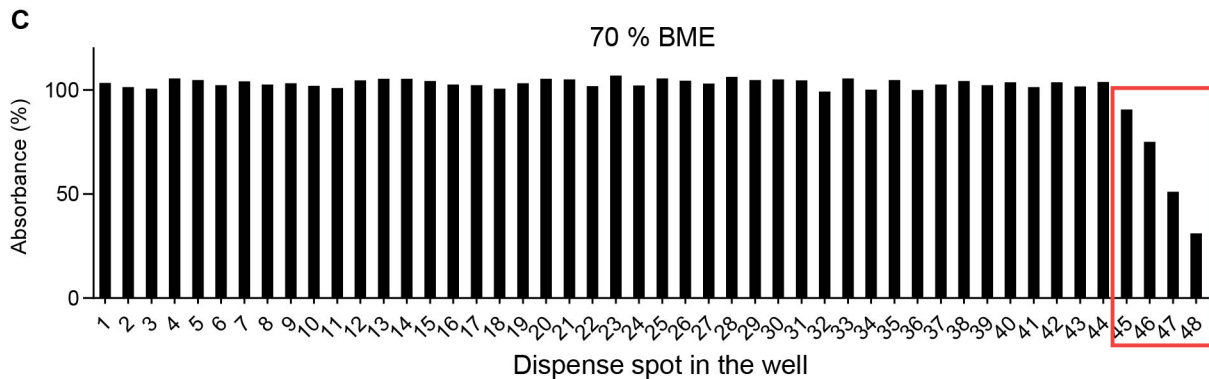

Supplement: Supplementary file 1 [file ijms-24-01006-s001.zip › Figure S1. Additional required dispense volume of ASFA SPOTTER V6.pdf]
